# Targeted Hybrid Nanocarriers as Co-Delivery Systems for Enhanced Cancer Therapy

**DOI:** 10.34172/apb.2024.046

**Published:** 2024-05-15

**Authors:** Joan Onyebuchi Erebor, Elizabeth Oladoyin Agboluaje, Ava M. Perkins, Megha Krishnakumar, Ndidi Ngwuluka

**Affiliations:** ^1^MAFEREB, LLC, 46701 Commerce Center Dr. Plymouth, MI 48170-2475 USA.; ^2^Department of Pharmaceutical and Biomedical Sciences University of Georgia, 250 W. Green Street Athens, Georgia 30602- 5036 USA.; ^3^Department of Pharmacy Practice, College of Pharmacy and Pharmaceutical Sciences, The University of Toledo 3000 Arlington Ave, Toledo, OH 43614-2595 USA.; ^4^Catalent Pharma Solutions, 7330 Carroll Road, San Diego, California 92121-2363 USA.; ^5^Department of Pharmaceutics, Faculty of Pharmacy, University of Jos, Pharmaceutical Sciences Gate, Bauchi Rd, 930001, Jos, Plateau State, Nigeria.

**Keywords:** Cancer therapy, Hybrid nanocarriers, Co-delivery, Drug delivery, Targeted, Nanoparticles

## Abstract

Hybrid nanocarriers have realized a growing interest in drug delivery research because of the potential of being able to treat, manage or cure diseases that previously had limited therapy or cure. Cancer is currently considered the second leading cause of death globally. This makes cancer therapy a major focus in terms of the need for efficacious and safe drug formulations that can be used to reduce the rate of morbidity and mortality globally. The major challenge encountered over the years with cancer chemotherapy is the non-selectivity of anticancer drugs, leading to severe adverse effects in patients. Multidrug resistance has also resulted in treatment failure in cancer chemotherapy over the years. Hybrid nanocarriers can be targeted to the site and offer co-delivery of two or more chemotherapeutics, thus leading to synergistic or additive results. This makes hybrid nanocarriers an extremely attractive type of drug delivery system for cancer therapy. Hybrid nanocarrier systems are also attracting attention as possible non-viral gene vectors that could have a higher level of transfection, and be efficacious, with the added advantage of being safer than viral vectors in clinical settings. An extensive review of various aspects of hybrid nanocarriers was discussed in this paper. It is envisaged that in the future, metastatic cancers, multi-drug resistant cancers, and low prognosis cancers like pancreatic cancers, will have a lasting solution via hybrid nanocarrier formulations with targeted co-delivery of therapeutics.

## Introduction

 Cancer therapeutics has the major challenge of non-selective cellular uptake for cancer cells in cancer therapy. Over the years, efficacious cancer treatment with low side effects on normal tissues has been quite difficult to achieve. This has resulted in poor prognosis in some types of cancer despite the wide range of cancer treatments currently available. The factors influencing the therapeutic outcome are multiple, including the nature of the disease, the actual stage of the disease, multidrug resistance, the physiological makeup of the human body, and individual human idiosyncrasies. Nanomedicines facilitated a shift from controlled release to a more targeted form of drug delivery with the focus on nanocarriers as possible drug delivery vehicles to various cancers such as very aggressive pancreatic cancer disease with a five-year survival rate of less than 5%, ovarian and breast cancer, to ensure selective, preferential accumulation of larger payloads within tumor sites,^1,2^ increased efficacy and increased safety with fewer side effects in drug delivery.^3^ Targeted cancer therapy using nanocarriers has gained traction and the Web of Science recorded almost 8000 peer-reviewed publications in the field of nanomedicine between 2015 and 2019.

 A hybrid nanocarrier can be defined as conjugates of organic and/or inorganic materials that were formulated by distinct methods to synergistically combine the advantages of the materials.^4^ Hybrid nanocarriers in drug delivery are unique nanocarriers that are aimed at harnessing the advantages of two or more nanocarriers and over the drawbacks of the individual nanocarrier systems to form a unique new nanocarrier structure that can be more efficacious and still safe.^5^ The ability to co-deliver different types of therapeutic agents in one formulation is also an added advantage of hybrid nanocarriers, especially in a disease such as cancer that has such a diverse heterogenous group of diseases.^6^ Cancer killed 9.9 million people globally in 2020 minus the cases due to non-melanoma skin cancer, with lung cancer contributing to 18% of cancer-related deaths globally amongst both males and females.^7^ Breast cancer is the most common cancer contributing to 11.7% of the 19.2 million new global cancer cases. The American Cancer Society projects that in 2023, there will be 1.95 million new cases and about 600 000 cancer-related deaths in the United States.^8^ These statistics suggest that there is a need for more translational research for there to be a substantial decrease in cancer morbidity and mortality globally and targeted hybrid nanocarriers seem to be a probable answer.

## Prevalent cancer treatment methods and their challenges

 Cancer has different treatments developed, including surgery, chemotherapy, radiation therapy, immune therapy, hormone therapy, and targeted therapy.^9^ Cancer was previously treated using surgical removal of the tissues so that it does not spread to other tissues. Surgery works best for solid tumors contained in one area.^10^ Chemotherapy destroys cancer cells anywhere in the body including metastases. Anticancer drugs administered via different pathways are either used alone or in combination to achieve better treatment efficiency.^9,11^ Radiation therapy is the use of radioactive substances and the application of high doses of radiation to kill cancer cells and shrink tumors for local treatment. Immunotherapy triggers own immune system or provide what it needs to kill tumor cells throughout the whole body. Hormone therapy is a treatment that slows or stops the growth of breast and prostate cancers that uses hormones to grow and are selective at the site of action, preventing early cancers.

 Targeted therapy are drugs that target specific changes in cancer cells that help them grow, divide, and spread thereby affecting normal tissues less.^9^ Chemotherapy possesses some advantages over radiation and surgery, and these include ease of administration, less invasive, and age-independent clinical outcome. The complexity of cancer limits the efficacy of a single anti-cancer agent which may display inefficient inhibition of tumor growth and as a result, cancer therapy is usually a concurrent administration of multiple anti-cancer agents. When the drugs are combined, positive effects such as elevated tumor inhibition efficiency, enhanced sensitivity of tumors to therapeutic agents, a reduced dose of toxic drugs, decreased adverse effects and prolonged survival are envisaged and possibly experienced. Combination therapy is a more effective approach to chemotherapy. The five most common cancers in both sexes in 2020 are breast, lung, colorectal, prostate, and stomach cancers.^7^ Various drugs that could be combined as first-line therapies found in the literature include cisplatin, paclitaxel (PTX), docetaxel, bevacizumab, carboplatin (CBP), topotecan, gemcitabine, fluorouracil, leucovorin, irinotecan, leucovorin, and oxaliplatin.^12,13^ Further discussion of the various chemotherapeutics that are available will be discussed later on. The conventional and available therapies due to wide distribution in the body precipitate adverse effects and destroy both cancerous and normal cells, thus more targeted delivery is needed.

## Targeting nanocarriers in drug delivery

 Targeting in drug delivery focuses on the ability of a therapeutic agent to act at the site of action with little or no activity on non-target tissues. The ability to selectively increase the concentration of the drug at the desired site of action while concurrently reducing the occurrence of activity on normal tissues, makes targeted nanomedicines very desirable as compared to non-targeted drug delivery systems, especially in cancer treatment. At present, this is achieved by delivery systems, either via passive or active targeting.^14^

###  Passive targeting

**Figure 1:**
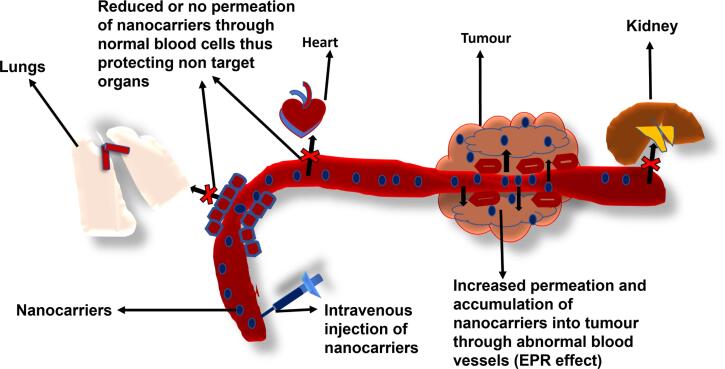
Schematic representation of passive targeting of cancer cells via the enhanced permeability and retention effect (EPR)^23^

 Passive targeting refers to a situation where particles preferentially accumulate inside the interstitial space of cells. Passive targeting has been observed to occur in various situations: by the (Mononuclear Phagocytic System) MPS combined with the blood and lymphatic vessels in the body, through changes in local physiological conditions in the body such as a reduction or an increase in pH or increased levels of enzymes, or via the most commonly reported passive targeting; enhanced permeability and retention (EPR) effect.^15^ It has been observed that there is an abnormally high porosity in the vasculature of cancer cells due to the excessive production of various vascular mediators and cytokines, such as bradykinin and vascular endothelial growth factor (VEGF) in cancer cells. This causes nanoparticles and macromolecular anticancer agents to preferentially accumulate more in cancer cells than normal cells and hence exert their cytotoxic effect with higher specificity. This mechanism is referred to as the EPR effect (Figure 1). PEGylation of nanocarriers has been shown to increase the accumulation of nanomedicine delivery in intratumor sites via the EPR effect *in vivo*.^16^ PEGylation of nanocarriers has been demonstrated to lead to an increase in therapeutic efficacy via enhanced permeability *in vivo* by increasing the circulation time of the nanocarriers, thereby leading to increased efficacy *in vivo* as demonstrated in triple-negative breast cancer (TNBC) therapy.^17^ The coating of the first FDA-approved medicine; PEGylated liposomal doxorubicin (DOX) HCl injection (Doxil®) has been proven to cause a reduction in the extent of uptake of the DOX liposomal formulation by eluding recognition and elimination by the reticuloendothelial system (RES).^3,18^ This becomes more relevant, especially in PEGylated hybrid nanocarriers maximizing their ability for passive targeting via the EPR effect. There are several recent studies that employ EPR to co-deliver therapeutics such as the metal-polymer hybrid nanoparticle system fabricated by Ghorbani and colleagues to co-deliver DOX and 6-mercaptopurine,^19^ lipid-polymer hybrid nanoparticle system synthesized by Wang and colleagues to co-deliver cisplatin and vinorelbine for the treatment of non-small cell lung cancer,^20^ and the metal-dendrimer hybrid nanoparticle system assembled by Lin and colleagues to co-deliver gemcitabine and miR-21 inhibitor for the treatment of pancreatic cancer.^21^ All nanocarrier systems demonstrated ideal physicochemical properties to effectively co-deliver the respective therapeutic agents to targeted cancerous cells and showed negligible activity in healthy cells, deeming this mechanism of delivery to be highly desired for cancer treatment. Although passive targeting has a lot of advantages, it has several challenges as well. The EPR effect is very dependent on several factors such as nanocarrier size being less than 500 nm, circulation time, intratumor pressure, and degree of heterogeneity. This can be explained by the fact that passive targeting is a slow process and the EPR effect may not occur homogeneously even within individual tumors.^22^

###  Active targeting

 The concept of active targeting was first conceptualized by Paul Ehrlich with his idea of the “magic bullet”. “The magic bullet” ideology has to do with the idea that when a drug is administered, it should go directly to the required site of action without causing damage to other cells or tissues.^24^ Active targeted drug delivery systems comprise three main constituents: the targeting moiety, the nanocarrier, and the drug or therapeutic agent to be released at the site.^13^ The targeting moiety or ligand grants hybrid nanocarriers the capacity to recognize specific antigens or cell membrane receptors and bind to them. This enables targeted hybrid nanocarriers to deliver chemotherapeutics to selectively targeted tissues and organs, and hence ensure reduced toxicity of delivered chemotherapeutics.^9^ Ligands that have been used for active targeting include various proteins, peptides, polysaccharides, and small biomolecules.^25^ Commonly used ligands include transferrin, folic acid, and hyaluronic acid.^26-28^ In recent studies, Lakkadwala and Singh fabricated a lipid-polymer nanocarrier system incorporating transferrin to co-deliver DOX and erlotinib to actively target brain endothelial (bEnd.3) cells,^29^ Liu and colleagues synthesized a lipid-polymer nanocarrier system incorporating folic acid to co-deliver daunorubicin and homoharringtonine to enhance the therapeutic effect on acute myeloid leukemia by actively targeting HL60 and K562 cells,^30^ while Boafo and colleagues assembled a lipid-polymer nanocarrier system including hyaluronic acid as a ligand to co-deliver daunorubicin and cytarabine to CD44 receptors that are overexpressed in cancer cells.^31^ Targeted hybrid nanocarriers utilized as non-viral vectors for gene delivery and delivering small interfering RNA (siRNA) to cancer cells have also been shown to lead to increased gene expression and occurrence of gene silencing respectively.^14,15,28^ Pancreatic cancer therapy using select biomarkers and targeted hybrid nanoparticles has been proposed as a solution due to the poor prognosis observed in patients that have been diagnosed to have pancreatic cancer.^16^

## Types of hybrid nanocarrier systems

**Figure 2:**
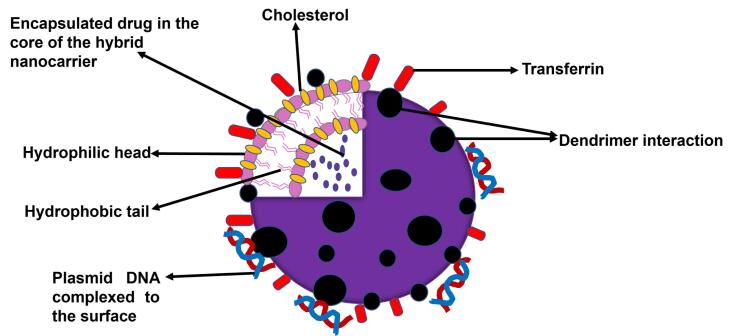
Schematic Structure of Transferrin of a targeted lipid–dendrimer hybrid nanocarrier particle with co-delivery of a drug and gene^23^

 Hybridization of two or more nanocarrier delivery systems has been demonstrated by various researchers to generate new structures that have enhanced the delivery of various cancer chemotherapeutics.^28^ Targeted hybrid nanocarriers have been generating a lot of interest due to the postulation that they can co-deliver more than one anticancer therapeutic agent and hence cause an additive or a synergistic effect. The concept of combining a polymer-based system and a lipid-based system to form a hybrid has been a huge breakthrough in the world of science and can be used to deliver drugs, genes, and diagnostic materials. Nanocarriers systems such as liposomes, polymers, lipids, niosomes, solid lipid nanoparticles, dendrimers, hydrogels, gold nanocarriers, and silica nanocarriers are examples of individual drug delivery systems that have been utilized to form hybrid nanocarrier systems. Based on the remarkably diverse materials and structures that make the different types of hybrid nanocarriers, various ways of classifying them exist.^28,32^ Classification of the types of hybrid nanocarriers that have been investigated is mainly based on their source materials (Table 1) or their formulation processes.^32,33^ Other miscellaneous subcategories also exist. Based on the classification shown in Table 1, the polymers and biomolecules or small molecule organics class are the most investigated hybrid nanocarriers especially the lipid-polymer and the lipid-dendrimer hybrid nanocarriers.^28,33^ A schematic representation of a lipid-dendrimer hybrid nanocarrier particle is shown in Figure 2.

**Table 1:** Classification of Hybrid nanocarriers based on their material composition

**Classification**	**Examples**	**References**
Polymers and biomolecules Or small molecule organics	Lipid-polymer hybrid nanoparticles	^34^
	Lipid-dendrimer hybrid nanoparticles	^35^
	Hybrid PLGA nanoparticle	^36^
	Folate-targeted lipid chitosan hybrid nanoparticles	^37^
	Polymeric hybrid micelles (TSP-TN)	^38^
	Lipid-Chitosan Hybrid Nanoparticles.	^39,40^
Polymers and metals	Hybrid Gold nanoparticles with PEG	^41,42^
Metal or metal alloy materials	Hybrid gold-iron oxide nanoparticles Dendrimer–gold nanoparticle hybrids	^43^
Metals and nonmetal inorganics	Hybrid hydrogels of α-cyclodextrin and polyethylene-modified gold nanocrystals	^32^
Nonmetal inorganics	Lipid-coated mesoporous silica hybrid nanoparticles	^44^
	PEI with mesoporous silica nanoparticles	^45^
	Hybrid Mesoporous–Microporous Nanocarriers for MDR	^46^

###  Lipid-polymer hybrid nanocarrier systems

 Lipid-polymer hybrid nanocarrier systems consist of mainly two nanocarrier systems: the lipid and the polymeric systems.^47^ The lipid-polymer hybridized systems are biocompatible and stable and can be modified in so many ways to deliver therapeutic agents.^33,47^ This makes them an extremely attractive platform for the next generation of drug delivery systems for cancer therapy. This class of hybrid nanocarriers has various sub-classifications based on their structure and method of formulation.^48^ Lipid-polymer hybrid nanocarrier systems can be classified based on their structure into Monolithic lipid polymer hybrid systems and Core-shell type systems.^35^

####  Monolithic lipid polymer hybrid system

 This is a type of system in which lipid molecules are uniformly dispersed within a polymeric matrix. Cai et al employed the use of a monolithic lipid polymer hybrid system in preparing a negatively charged monolithic system using pectin sulfate-loaded lipid polymer nanoparticles and rhamnolipid and phospholipids.^49^

####  Core-shell type systems

*Polymer core-lipid shell: *The polymer core-lipid shell system, also called lipid-coated nanoparticles, has a polymer core with several layers of lipid layers around it. Hybridization provides benefits like the immediate release of the drug and controllable particle size.^50^ To achieve better serum stability and the ability to load various agents efficiently, charged phospholipids can be employed. Pancreatic cancer patients who in the past have been treated with gemcitabine usually had a survival rate of only an additional five to seven and a half months on average. Zhao et al developed biocompatible lipid-polymer hybrid nanoparticles for the co-delivery of HIF1a siRNA (si-HIF1a) and Gemcitabine for the treatment of pancreatic cancer. Treatment of panc-1 subcutaneous xenografts *in vivo* with these lipid-polymer hybrid nanoparticles showed a better survival rate with less drug resistance in BALB/c nude mice. Gemcitabine was loaded into the hydrophilic core of the cationic polymer while si-HIF1a was absorbed on the surface. The siRNA was protected from immune system recognition and serum destruction was prevented by the protective ability of the pegylated lipid bilayer shell. It was observed the nanocarrier was stable and was able to circulate for up to three hours in the bloodstream and this led to better anticancer properties.^51^

*Hollow core lipid polymer lipid system: *In this system, there is an innermost layer of the hollow core with a lipid layer surrounded by a polymeric layer.^47^ This system was used to encapsulate up to 80% of siRNA within the innermost hollow core that has the cationic lipid. The ability to deliver siRNA to specific tumor growths has a great advantage in the treatment of cancer. Also, small drug molecules could be co-delivered to achieve a synergistic therapeutic action for cancers that has multi-resistant properties.^52^

*Biomimetic lipid-polymer hybrid system (BLPHS): *The BLPHS type of system can also be described as erythrocyte membrane camouflaged polymer nanoparticles. The nanoparticles are coated with red blood cell membranes.^37^ The biomimetic lipid-polymer hybrid system has been shown to circulate for up to 39.6 hours in the bloodstream before being eliminated while polyethylene glycol showed 15.8 hours of circulation time.^47^ A recent study successfully loaded DOX into a folate-functionalized erythrocyte membrane vesicle coated with magnetic nanoparticles for ovarian cancer treatment. The biomimetic nanocarrier showed improved anticancer properties in the treatment of ovarian cancer.^53^ Xie and colleagues successfully loaded curcumin into porous poly (lactic-co-glycolic acid) nanoparticles with red cell membranes conjugated to the surface. The use of red blood cells to hide the nanoparticles aided the anticancer efficiency of curcumin.^54^

*Polymer-caged liposomes system (PCLS): *These are very stable systems in which polymers are placed on the surface of liposomes. A polymer-anchored liposome system with a diameter of 123 ± 11 nm was designed by Aoki et al.^55^ This system could be activated with temperature and was shown to be stable for 8 hours after administration. This increases the anti-cancer effect. At 40 °C, the hydrophilic polymer anchored on the liposome changed to hydrophobic and a contrast agent that can be activated.^47^ The ability to use this system for *in vivo* imaging provides a temperature-influenced method of cancer diagnosis and treatment. This design is minimally invasive for cancer treatment.^55^

###  Metal hybrid nanocarrier systems

 Metal hybrid nanocarrier systems have several subcategories containing differing nanocarrier systems including metal and either polymer, lipids,^56^ metal alloy materials, or non-metal organic materials (Table 1). They are typically synthesized with metals including but not limited to silver, aluminum, iron, gold, silica, copper, zinc, magnesium, cerium, titanium, platinum, or thallium. Once inside the cell, the nanoparticles trigger a cascade of reactive oxidative species and release metal ions, resulting in the destruction of disulfide bridges and the activation of several signaling pathways leading to cell death such as apoptosis, autophagy, and programmed necrosis of cancerous cells.^57^ Metal hybrid nanocarrier systems have shown promise in preventing multidrug resistance, increasing stability and half-life, and improving biodistribution and passive or active targeting.^58^

###  Non-metal inorganic hybrid nanocarrier systems

 Non-metal inorganic hybrid nanocarrier systems are broader, less common systems that contain components that are neither metal nor organic with the goal of improving drug delivery. Mesoporous silica nanoparticles (MSNs), for example, are non-metal inorganic materials used to increase the safety and efficacy of drug delivery by preventing mechanical, thermal, and biological degradation of encapsulation technology.^59^ In a study by Paris and colleagues, MSNs were used as drug carriers and as nuclei for the generation of inertial cavitation to improve drug delivery and penetration for cancer therapy.^60^ Zheng and coworkers used a similar method, using MSNs to deliver sorafenib (SOR) and VEGF-targeted siRNA for asialoglycoprotein receptor-mediated targeted hepatocellular carcinoma.^61^

## Synthesis of hybrid nanocarrier systems

 Methods of formulating hybrid nanocarriers vary widely. Examples include microﬂuidic synthesis emulsification-solvent evaporation, emulsification-solvent diffusion, layer-by-layer (LbL) synthesis, and nanoprecipitation. Hybrid nanocarriers can be formulated either by single-step process, multiple-step process, or non-conventional process. Multi-step has been recognized as the process that can be used to control the lipid-polymer ratio best for lipid-polymers hybrid nanocarriers. The various methods of formulation are discussed:

###  Microfluidic method

 The synthesis of hybrid nanocarriers using a microfluidic approach consists of basic mixing strategies such as hydrodynamic focusing and microstructure mixing enhancement, bottom-up synthesis methods to ensure uniform size and distribution, adjustable multilayer structures, and ideal physicochemical properties. Hydrodynamic focusing occurs in a three-inlet channel microfluidic device, and mixing time is improved with the proper solutions, flow rates, and mixing channel length. Mixing efficiency can also be improved by adopting microstructures such as straight microchannels, curved microchannels, double spiral microchannels, and herringbone mixers. Both techniques enhance the formulation process so that mixing time is shorter than nucleation, resulting in the formation of either micro or nano-sized particles. These strategies ensure that bottom-up methods, such as self-assembly, nanoprecipitation, sol-gel, reduction,^62^ and polymerization build nanoparticles from their simplest form for easy surface modification, adjustable rigidity, controllable LbL structures, and consistent size.^63^ Yan and colleagues fabricated a non-metal inorganic nanocarrier system with mesoporous silica, polystyrene sulfonate, DOX, and PTX using microfluidic techniques. It was found that this technique improved drug release with 70% release after 20 hours, increased the selectivity of breast cancer cells, and resulted in negligible activity in healthy breast cells.^64^

###  Emulsification-solvent evaporation method

 The synthesis of nanoparticles using the emulsification-solvent evaporation method is well-recognized in the formation of hybrid nanocarrier systems. The mechanism consists of an organic phase containing a drug and lipophilic surfactant, dissolving it in a volatile organic solvent, and adding it to the aqueous phase containing a water-soluble surfactant. After emulsification using stirring and high-pressure homogenization, the solvent evaporates, creating nanoparticles.^65^ Babos and coworkers used the emulsification-solvent evaporation method in the creation of lipid-polymer hybrid nanocarriers to co-deliver SOR and DOX for the treatment of hepatocellular carcinoma.^66^ Their formulation yielded a particle size of 177.2 nm, PDI of 0.076, entrapment efficiency of 69% for DOX and 88% for SOR, and drug loading of 4.17% for DOX and 5.31% for SOR. Additionally, the system demonstrated higher cellular uptake and higher cytotoxicity in HT-29 cancer cells.^66^

###  Emulsification-solvent diffusion method

 The emulsification-solvent diffusion method is one of the most commonly used methods in the preparation of hybrid nanocarrier systems.^65^ In this method, an organic phase consisting of a drug and lipophilic surfactant is dissolved in a water-miscible solvent and added to the aqueous phase consisting of a water-soluble surfactant. Once emulsified after stirring and high-pressure homogenization, the emulsion is further diluted with water, and the organic solvent diffuses, resulting in the formation of nanoparticles.^67^ This technique is favorable in the formulation of hybrid nanocarrier systems as it produces better encapsulation capacity, higher reproducibility, and precise control of particle size.^65^ Zhang and colleagues fabricated a nanocarrier system using trastuzumab-coated lipid-polymer hybrid nanoparticles composed of poly (D, L-lactide-co-glycolide), polyethylenimine, and other lipids with docetaxel using the emulsification-solvent diffusion method for the treatment of breast cancer.^68^ In the study, the mean particle size was 217.4 nm, the zeta potential was 0.056 mV, the PDI was 0.116, the entrapment efficiency was 31.27%, and the hybrid nanocarrier system was more toxic to HER2-positive BT474 cells compared to blank lipid-polymer nanoparticles (LPNs).^68^

###  Layer-by-layer method

 The LbL technique is another method of synthesis for hybrid nanocarrier systems. In this technique, the first layer is formed by immersing a charged nanoparticulate in a polyelectrolyte solution consisting of an opposingly charged polymer. Excess polymer is then removed either by washing or centrifugation before the addition of the second layer of an oppositely charged polymer from the first layer. This process is repeated until the appropriate coating is achieved.^69^ LbL assembly is advantageous in that it can incorporate and preserve biological activity, coat large surface areas efficiently, and withstand typical changes in temperature, pH, and ionic strength.^70^ Zhang et al synthesized a metal-polymer hybrid nanocarrier system consisting of platinum complex and chitosan for the co-delivery of cisplatin and gemcitabine as a treatment for lung carcinoma using LbL assembly.^71^ Through this method of synthesis, particle size was 187 nm, the zeta potential was -21 mV, and entrapment efficacy of 90%; both drugs were released in a sustained manner, and high cytotoxicity was observed in NCl-H460 cells.^71^ Kabary and colleagues also formulated a hybrid nanocarrier system using lactoferrin and hyaluronic acid to co-deliver rapamycin and berberine to treat lung carcinoma using LbL synthesis.^72^ They concluded that their system was superior in delivering both drugs with physicochemical characteristics such as a particle size of 250.5 nm, a zeta potential of -18.5 mV, controlled release of both therapeutics, and enhanced toxicity against A549 lung cancer cells.^72^

###  Nanoprecipitation method

 There are three widely used methods in nanoprecipitation synthesis including traditional nanoprecipitation, flash nanoprecipitation, and microfluidic-based nanoprecipitation. Because microfluidic techniques are previously discussed, this section will only focus on the traditional and flash methods. In traditional nanoprecipitation, an organic and aqueous phase is combined under traditional mixing to produce nanoparticles. This method is typically quick, easy, and cheap to operate but is often hard to control resulting in large and inconsistent particle sizes.^73^ In flash nanoprecipitation, mixing devices such as a confined impinging jet (CIJ) mixer or a multi-inlet mixer (MIV) rapidly create supersaturated conditions that lead to the precipitation and encapsulation of nanoparticles.^74^ Preparing nanoparticles using this method is fast, has good reproducibility, and has high drug loading capability, but the particle stability, or lack thereof, may not be suitable for some applications. Gao and colleagues explored the effectiveness of a nanocarrier system consisting of PLGA and D-alpha-tocopherol polyethylene glycol 1000 succinate to co-deliver docetaxel and salinomycin in the targeting of breast cancer and stem cells through nanoprecipitation synthesis.^75^ The specific technique of nanoprecipitation was not specified, but they discovered that the particle size of the system was 73.83 nm, a PDI of 0.193, a zeta potential of -25.7 mV, entrapment efficiency of 82.3%, drug loading of 4.12%, and enhanced cytotoxicity against MCF-7-MS cells.^75^

## Advantages of targeted hybrid nanocarriers

 The major idea behind the use of targeted hybrid nanocarriers was to combine the advantages and improve on the disadvantages of the two structural components for a better design, that can enhance the therapeutic effect of the drug being delivered.^50^ Their capacity to deliver a combination of anticancer therapies with different mechanisms of action would provide a solution to the challenge that the heterogeneity, unique microenvironment, and physiological structure of solid tumors possess.^76^ Multidrug resistance (MDR) in cancer cells has also been overcome by utilizing targeted hybrid nanocarriers *in vivo*.^9,46^ Lipid-polymer hybrid nanocarrier systems have several advantages over the usual lipid system as seen in liposomes and polymeric systems. The advantages of lipid-polymer hybrid nanocarriers stem from their design; a biodegradable polymeric core that houses the therapeutic agent which is now surrounded by two layers of lipid (a middle layer and an outer layer). The outermost lipid layer can be coated with polyethylene glycol and can thus increase circulation time in the body by giving a stealth effect to the whole nanocarrier. The polymeric core enables these improvements; mechanical stability on storage, structural strength, serum stability, targeting, and preferable release profile due to its framework.^47^ Lipid-based systems have better biocompatibility, bioavailability, and drug-loading capacity.^47^ The polyethylene end group can be modified on the surface to produce non-immunogenic hybrid nanocarriers that can exhibit both passive and active targeting. With the invention of targeted hybrid nanocarriers, siRNA can be delivered to target cells. This shows huge potential in the treatment of MDR cancer by co-delivery of siRNA and drug molecules using hybrid nanocarriers.^52^ Drug toxicity gets decreased by encapsulation into the hybrid nanocarrier and this can also increase the half-life of a drug.^77^ This advantage has been noticed with liposomal DOX which causes cardiac toxicity. Several drugs for the treatment of cancer have been formulated by hybridization of protein-polysaccharide. Hybridization of these drugs can be achieved by electrostatic complexation, electrospinning, and chemical conjugation. Some of the drugs including docetaxel were formulated with the hybridization of albumin and carboxymethylcellulose. Enhanced active targeting was noticed which improved the antitumor activity of the docetaxel. Doxorubicin was formulated with the hybridization of albumin and chitosan and an enhanced anti-tumor effect was observed in HepG2 hepatocellular carcinoma cell line. Higher anti-tumor activity was seen in A549 and H460 lung adenocarcinoma cell lines when bovine serum albumin and chitosan were used as the hybrid nanocarriers compared to when albumin alone was used. Also, DOX and ellagic acid showed better absorption and cytotoxic effect when lactoferrin and chondroitin were used as nanocarriers.^78^ Oleshkevich and colleagues reported that magnetic nanoparticles nanohybrids coated with m-carboranylphosphinate (1-MNPs) for the treatment of human brain endothelial cells (hCMEC/D3) and glioblastoma multiform A172 cell line led to increased cell penetration and anti-tumor activity without cellular toxicity observed in the cells.^79^

## Challenges of targeted hybrid nanocarriers

 Targeted hybrid nanocarriers can be capital and labor-intensive. There have been issues of scalability and when this is successfully done, the chemotherapeutic agents might be too expensive for most people to afford it. PEGylating the outermost lipid layer was done to overcome the liposomal physiological instability, but this has shown an increase in skin toxicity.^80^ When it comes to delivering more than one drug together, it can be challenging to develop methods and determine whether the drugs will produce the same synergistic effect during *in vivo* tests as they show during *in vitro* tests. Targeting cells specifically to reduce toxicity is a very complex design that requires a lot of modeling.^77^ Also, the pharmacokinetics and biocompatibility of targeted hybrid nanocarrier can be challenging to optimize.^81^

## Classes of cancer therapeutics and their mechanisms of actions

 Some classes of anticancer agents and their mechanisms of action as listed in Table 2 are further defined and elaborated on in this section.

**Table 2:** Classes of cancer therapeutics and their mechanisms of actions

**Classes of drugs**	**Examples in each Class**	**Target site**	**Mechanism of action**	**Organ/cancer**
Alkylating agents*	Altretamine, bendamustine, busulfan, carboplatin, carmustine, chlorambucil, cisplatin, cyclophosphamide, dacarbazine, ifosfamide, melphalan, oxaliplatin, temozolomide	Nucleus	Destroys DNA and interferes with cellular mitosis	Breast, lung, ovarian, leukemia, lymphoma, Hodgkin disease, multiple myeloma, and sarcoma
Anti-microtubule agents*	1. Vinca alkaloids: vinblastine, vincristine, vinorelbine 2.Taxanes: cabazitaxel, docetaxel, nab-paclitaxel, paclitaxel	Microtubules	Prevent the formation of microtubules. Inhibit the microtubule disassembly	Breast, lung, myelomas, lymphomas, leukemias
Anti-metabolites*	Azacitidine, clofarabine, cytarabine, decitabine, floxuridine, gemcitabine, 5-fluorouracil, 6-mercaptopurine, hydroxyurea, methotrexate, thioguanine, trifluridine/tipiracil combo	Nucleus	Disrupts DNA and RNA formation by acting as a substitute for the normal building blocks of RNA and DNA	Breast, ovary, and intestinal tract
Topoisomerase inhibitors*	Trinotecan, topotecan, irinotecan, etoposide, teniposide	Nucleus	Impair the activity of topoisomerase and disrupt catalytic turnover	Colon, lung, intestinal tract, pancreas
Cytotoxic antibiotics*	Doxorubicin, daunorubicin, epirubicin, idarubicin, valrubicin, bleomycin, dactinomycin, mitomycin-C	Nucleus	Bind to DNA, preventing the cancer cells from growing and multiplying	Sarcomas,
Monoclonal antibodies*	Bevacizumab, pertuzumab, rituximab trastuzumab	Protein receptors	Identify specific proteins on cancer cells and lock in or attach to them and in the process, kill cancer cells by preventing the growth/division of cells	Breast, brain, colorectal, lung, prostate, melanoma
PARP (poly-ADP ribose polymerase) *	Olaparib Talazoparib	PARP	Inhibits PARP from its repair activity in cancer cells leading to cell death	Ovarian, fallopian tube, peritoneum, metastatic breast cancer^82^
PI3K/AKT/mTOR pathway Inhibitors	Duvelisib	PI3K	Targets both the δ isoform and γ isoform of PI3K. The δ isoform is important for the proliferation and survival of cells. The γ isoform receives pro-inflammatory responses from the microenvironment and is involved in cytokine signaling.	Lymphocytic leukemia, follicular lymphoma^83,84^
	Everolimus	mTOR	Binds to KKBBP12 with high affinity to an inhibitor of the MTOR. This, therefore, prevents the downstream signaling needed for cell growth and proliferation.	Metastatic breast cancer^85^
	Capivasertib	AKT	Inhibits the AKT pathway and therefore the phosphorylated pathway proteins (GSK3β, PRAS40, and S6) are hindered.	Metastatic breast cancer^86^

*Compiled from cancer.org; cancerresearchuk.org.

###  Alkylating agents 

 Alkylating agents prevent cells from reproducing by damaging DNA and are used to treat a variety of cancers and diseases. However, in rare cases, alkylating agents can cause leukemia due to damage to bone marrow, so it is preferred that treatment is used in low doses to mitigate further risk.^22^ Wan and colleagues fabricated a polymeric hybrid nanocarrier system for the co-delivery of cisplatin, an alkylating agent, and PTX, an anti-microtubule agent.^87^ In this study, the combination of drugs in the system displayed superior anti-tumor activity in A2780/CisR xenograft tumor and LCC-6-MDR orthotopic tumor models that are typical for human ovarian carcinoma and multidrug-resistant breast cancer, respectively.^87^ Wang and the team found promising results with a polymeric hybrid nanocarrier system for the delivery of cisplatin and PTX for the treatment of ovarian cancer, with strong synergistic effects on SKOV3 cells.^88^

###  Anti-microtubule agents 

 Anti-microtubule agents prevent cell growth by inhibiting mitosis by interfering with the microtubules.^89^ There are two main classes of anti-microtubules: vinca alkaloids and taxanes. While effective in treating cancers such as breast, lung, and melanomas, there are limitations to their use due to the severe side effects, causing a reduction in a dose that is too low to receive a therapeutic effect.^90^ Zhang and colleagues recently explored the efficacy of a lipid-polymer hybrid nanocarrier system to co-deliver docetaxel, an anti-microtubule agent, and resveratrol, an antioxidant.^91^ Ideal properties like particle size, drug release, cellular viability, and cytotoxicity demonstrated that drugs were efficient in synergistically inhibiting PC3 and DU145 cells that are commonly found in prostate cancer compared to docetaxel alone.^91^ Zafar and colleagues also found a hybrid lipid-polymer nanocarrier consisting of docetaxel- and thymoquinone-loaded chitosan-grafted lipid nanocapsules to be highly cytotoxic against MCF-7 and MDA-MB-231 drug-resistant breast cancer cells.^92^

###  Antimetabolite

 Antimetabolites prevent cells from reproducing by interfering with the production of a major nucleotide metabolite by acting as RNA and DNA building blocks. They commonly treat leukemias, breast, ovary, and intestinal tract cancers, but also have a negative impact on normal cells such as bone marrow.^93^ Zhang and colleagues synthesized lipid-peptide hybrid nanoparticles for the co-delivery of gemcitabine (GEM), an antimetabolite, and PTX for the treatment of breast cancer.^94^ The prepared nanoparticles demonstrated physicochemical properties ideal for drug delivery such as a mean diameter of 85.1 nm, a positive zeta potential of 18.3 mV, high entrapment efficiency of 93.6% for GMP and 98.7% for PTX, and drug loading of 6.3% for GMP and 0.8% for PTX. Additionally, the system did a sufficient job of killing tumorous cells via apoptosis occurring 43.6% more within the hybrid nanocarrier system than the control sample. The expression also significantly decreased in B-cell lymphoma-2 and B-cell lymphoma-extra-large proteins.^94^ Liu and colleagues also discovered promising results when combining antimetabolite gemcitabine (GEM) with cisplatin (CDDP) using hybrid LPNs to potentially treat lung, breast, colon, and pancreatic cancer.^95^ The average particle size was 139.3 nm, PDI of 0.098, the zeta potential of -11.1 mV, drug loading of 2.46% for GEM and 1.48% for CDDP, and entrapment efficiency of 47.7% for GEM and 14.7 for CDDP. The system displayed sustained drug release behavior, increased cellular uptake, and effective cytotoxicity behavior to cancerous lung cells.^95^

###  Topoisomerase inhibitors

 Topoisomerase (TOP) inhibitors interfere with topoisomerase enzymes by blocking the ligation step of the cell cycle, leading to apoptosis.^96^ Common cancers that are treated with TOP inhibitors include certain leukemias, and lung, ovarian, gastrointestinal, and pancreatic cancers. Irinotecan is known to be beneficial to patients with metastatic colorectal cancer, however, the use of irinotecan is fraught with drug resistance, non-selective distribution, and adverse effects.^5^ It is envisaged that specific targeting of the tumor and co-delivery of irinotecan with another chemotherapeutic agent will improve the selective destruction of tumor cells and reduce adverse effects and resistance. Wang and colleagues designed and developed targeted ligand lipid-polymer hybrid nanoparticles for the co-delivery of plasmid DNA and irinotecan leveraging on the combined benefits of gene therapy and chemotherapy.^12^ Hyaluronic acid (HA) was employed as the ligand for targeting as HA has a high binding affinity to the CD44 receptors which are overexpressed on the surfaces of tumor cells. The targeted hybrid nanocarriers were fabricated by solvent-evaporation technique and thereafter, characterized. The average particle was 182.3 nm, with a polydispersity of 0.17, zeta potential of – 21.3 mV, entrapment efficiency of 81.5 %, and gene loading of 90.3 %. The targeted nanocarriers displayed more sustained release, higher cellular uptake, and better cell inhibition efficiency than the non-targeted hybrid nanocarriers. The targeted hybrid nanocarriers displayed impressive tumor inhibition efficacy and gene transfection efficiency suggesting synergistic therapeutic efficacy of co-delivered anti-cancer drug and gene co-delivered therapy.

###  Cytotoxic antibiotics 

 Cytotoxic antibiotics, or anti-tumor antibiotics, are derived from Streptomyces bacteria and interfere with DNA replication causing apoptosis. They treat a wide variety of cancers including leukemias, breast cancer, lymphoma, and a variety of metastatic cancers. Like the previous classes of drugs mentioned, cytotoxic antibiotics have the capability to target healthy cells as well as cancerous ones, including bone marrow.^97^ In a recent publication, Khademi and colleagues fabricated metal-polymer hybrid nanoparticles with chitosan and gold to co-deliver DOX, a cytotoxic antibiotic, and nucleolin aptamer to evaluate their effect on Forkhead box M1, a transcriptional protein associated with carcinogenesis, cell proliferation, and metastasis.^98^ In the study, the DOX-Apt-CS-gold nanoparticles (AuNPs) system was 60.4 nm in size, had a zeta potential of 20.7 mV, a high drug loading capacity with the molar ratio of aptamer-chitosan-gold nanoparticle to DOX being 1:6.6, and drug release of 57% over 72 hours. Additionally, cytotoxic activity against 4T1 and A549 cells and negligible activity was observed against non-targeted cells, further confirming that this combination therapy is an effective method for treating cancerous cells.^98^ Chen and colleagues also designed and developed polydopamine (PDA)-coated AuNPs for the targeted PH-responsive co-delivery of DOX and photothermal therapy to achieve a synergistic therapeutic outcome. The targeted hybrid nanocarrier was fabricated by dopamine (DA) polymerization on AuNPs surface followed by i-motif DNA and AS1411 aptamer arrangement on the hybrid nanocarrier. The i-motif DNA is PH responsive and can change into a C-quadruplex construct in an acidic area. Dox was loaded on the i-motif DNA while the Au was the photothermal agent.^99^ The characterization of this targeted hybrid nanocarrier showed that the average particle size for Au@PDA-AS141 was 180 ± 2.6 nm with a zeta potential that changed from -36.3mV to -18.1 ± 1.3 mV after modifying the hydroxyl group on the AuNPs. It was also observed that coating PDA on the AUNPs increased the loading efficiency of Dox and the in-vivo anticancer study showed that the tumor volume for DAu@PDA-AS141 was close to zero for the group that had near-infrared irradiation. DAu@PDA-AS141 NPs displayed impressive tumor inhibition efficacy with excellent biocompatibility and cell viability.^99^

###  Monoclonal antibodies 

 Monoclonal antibodies are targeted drug therapies that bind to cell surface antigens that are associated with the growth and differentiation of cancer cells.^78^ Once bound to the target antigen, direct tumor cell death, immune-mediated tumor cell killing, vascular ablation and disruption of stromal interaction with cancer cells occurs depending on the mechanism, mAbs treat various cancers including leukemia, lymphoma, lung, breast, brain, and colorectal cancers.^100^ Ngamcherdtrakul and colleagues use a non-metal inorganic-polymer hybrid nanoparticle system with mesoporous silica and polyethyleneimine/polyethylene glycol using LbL assembly to co-deliver trastuzumab (an anti-HER2 antibody), siHER2, and docetaxel.^101^ Particle size ranged between 96.4 nm and 142 nm, PDI between 0.24 and 0.32, and drug loading between 0.04 and 1.71%. It was also found that the system was effective in treating breast cancer tumors in mice and displayed no signs of toxicity in the cell lines and blood of mice, showing great promise as a therapeutic agent for the treatment of breast cancer.^101^

###  Poly-ADP ribose polymerase inhibitors 

 Poly-ADP ribose polymerase (PARP) inhibitors cause apoptosis of cancer cells by inhibiting DNA repair pathways in homologous recombination-deficient cells and have recently been recognized for their high efficacy and low toxicity in treating ovarian cancer.^102^ Although showing promising qualities for treatment, off-target organ toxicity and drug resistance still need to be addressed and can be improved using hybrid nanocarrier systems.^103^ Mensah and colleagues used the LbL method to synthesize a hybrid nanocarrier system of olaparib and talazoparib with liposomal nanoparticles - 1,2-distearoyl-sn-glycero-3-phosphocholine, 1-palmitoyl-2-oleoyl-sn-glycero-3-phospho-(1′-rac-glycerol), and cholesterol (in a mass ratio of 56:39:5).^104^ The system was then encapsulated using cisplatin. Physicochemical properties include a particle size of 100nm, a zeta potential of -31 mV, and a PDI of 0.12. Additionally, the LbL polymeric liposomal NPs supported favorable clearance and biodistribution, longer bioavailability, and reduced adverse effects *in vivo* showing promising potential in the treatment of ovarian cancer.^104^

## Discussion

 Hybrid nanocarriers provide the ideal system to co-deliver cancer therapeutics due to their dual-component structure. The application of two core materials (such as lipid and polymer) enhances delivery and should the system be pH-responsive, it facilitates effective delivery into the tumors. In addition to pH responsiveness, conjugation of the nanoparticles with specific ligands can ensure specific or targeted delivery to specific sites. Biodegradable Lipid-polymer hybrid nanoparticles are a superior drug delivery system due to the combination of the benefits of liposomes and polymer nanoparticles such as superior biocompatibility, high drug loading, sustained release, and easy modification of targeting molecules including aptamers.^105^

 Wang fabricated CBP-PTX co-loaded folate-conjugated, pH-responsive LPNs for the treatment of cervical cancer.^12^ Folate (FA) was used as a receptor-specific ligand due to its high affinity for folate receptors which are over-expressed in diverse carcinomas. Characterization of the targeted hybrid nanocarriers revealed that the average size was 169.9 nm with a particle size distribution of 0.151, a Surface charge of 32.9 mV, and entrapment efficiencies of CBP and PTX were 83.1 % and 84.2 % respectively. The in vitro drug release studies displayed the pH-responsiveness of the system. At pH 5.5, over 80 % of CBP and PTX were released in 24 h while at pH 7.4, the same amount was released in 48 h. Cellular uptake efficiency was evaluated on Hela cells and it was found that the uptake efficiency of folate-conjugated nanocarriers (FA-CBP/PTX-LPNs) was more than the non-conjugated nanocarriers by 35.1%. Cytotoxicity revealed that there was improved cytotoxicity with the FA-CBP/PTX-LPNs compared to the free drugs. *In vivo* tissue distribution showed more concentration of FA-CBP/PTX-LPNs in the tumor than CBP/PTX-LPNs and free CBP/PTX. Such a system due to limited drug concentration in other tissues may reduce adverse effects of the cancer therapeutics and fewer complications. The co-delivery of the two drugs at the tumor site at the same time released over the same period may produce a synergic effect leading to better patient outcomes. Irinotecan is known to be beneficial to patients with metastatic colorectal cancer, however, the use of irinotecan is fraught with drug resistance, non-selective distribution, and adverse effects.^5^ It is envisaged that specific targeting of the tumor and co-delivery of irinotecan with another chemotherapeutic agent will improve the selective destruction of tumor cells and reduce adverse effects and resistance.

 Recurrence of cancer such as breast cancer after chemotherapy and radiation has been attributed to a subset of cells called cancer stem cells (CSCs) which will proliferate and self-renew to form new tumors. CSCs can withstand standard chemotherapy and radiotherapy, metastasize, and form new tumors. CSC could also emanate from non-CSCs in an epithelial-to-mesenchymal transition (EMT)-dependent process promoting tumor growth.^106^ Destruction of the CSCs and non-CSCs is paramount for effective cancer therapy and improvement of the quality of life of the patient. In a study, Yang and colleagues fabricated hyaluronic acid-coated lipoid-polymer nanoparticles for the co-delivery of PTX and curcumin,^44^ for the elimination of breast CSCs and non-CSCs to destroy breast tumors.^106^ HA was the targeting moiety used to target CD44 expressed on the breast cancer cells as HA specifically interacts with CD44 leading to enhance cellular uptake of HA-hybrid nanoparticles. The co-cancer therapeutics-loaded hybrid nanoparticles effectively enhanced the efficacy of breast tumor growth suppression by also eradicating both CSCs and non-CSCs. PTX-CUR-loaded HA-hybrid Nanoparticles displayed prolonged *in vivo* circulation, increased accumulation in breast tumors, the synergistic therapeutic effect of the two drugs, and eradication of CSCs and non-CSCs.

 The non-small cell lung cancer is one of the leading causes of cancer death and traditional chemotherapy is fraught with limitations such as poor cellular uptake, non-specificity, and adverse drug effects. Specific targeting of tumors cell will reduce adverse effects and improve clinical response. Aptamers are employed to enhance targeting as overexpressed receptors are the main targets of aptamers. Aptamers (APT) conjugated on lipid-polymer hybrid nanoparticles (LPHNs) reduce non-specific toxicity and enhance tumor targeting and cellular uptake. Wu and colleagues fabricated APT-LPHNs loaded with the prodrug of docetaxel (DTXp) and cisplatin (CDDP) for the management of non-small cell lung cancer.^105^ A comparative study of APT-DTXp/CDDP-LPHNs, DTXp/CDDP-LPHNs, APT-DTXp-LPHNs, APT-DTX-LPHNs, APT-CDDP-LPHNs, and free DTX/CDDP was undertaken and APT-DTXp/CDDP-LPHNs displayed a pronounced cytotoxicity, synergy antitumor effect, profound tumor inhibition ability compared to the other formulations.

 Thyroid cancer is the major endocrine malignancy and about 77 % of the diagnoses are in women.^107^ Thyroid cancer is the fifth most prevalent cancer in women in the United States and in 2015, there were 62 000 cases in both males and females.^108^ While there are patients with lower risk presenting benign thyroid nodules, and low mortality cases, there are aggressive malignancies (anaplastic thyroid cancer) requiring aggressive treatments. Both synthetic and natural drugs have been formulated for treatment employing various drug delivery systems, however, hybrid nanocarriers offer a remarkable delivery system that is multifunctional, encapsulating more drugs with different physicochemical properties that promote synergism, enhance targeting, and may combat resistance due to the different mechanisms of action of the drugs encapsulated. Massaro and colleagues fabricated multi-cavity halloysite-amphiphilic cyclodextrin hybrid nanotubes loaded with natural drugs, silibinin, and quercetin for uptake into thyroid cancer cells.^109^ In comparison with the free drugs, halloysite-cyclodextrin nanotubes exhibited improved cellular cytotoxicity and a synergistic effect was observed with the two drugs on an anaplastic thyroid cancer cell line. The hybrid nanotubes penetrated the cells and concentrated around the cell nucleus, an indication that the hybrid nanotubes could effectively be transported into living cells.^109^ Multi-mechanical actions of co-delivered cancer therapeutics have thus been found to be needed to provide an additive or synergistic effect in cancer therapy.

 Conventional chemotherapy employing free cancer therapeutics in combination is fraught with non-specific cytotoxicity of chemotherapeutic drugs, poor uptake of drugs in tumor cells, adverse systemic toxicity, and drug resistance. Co-delivery of cancer therapeutics in a single carrier system improves therapeutic efficacy by additive or synergistic effects and reduces multi-drug resistance. Drug resistance develops due to the temporal response of tumors to cancer therapeutics facilitated by the genetic diversity among tumors. In addition, alterations in the tumor microenvironment limit the absorption and delivery of drugs thereby precipitating resistance. Several ways of developing drug resistance include alterations in drug influx/efflux mechanisms, inactivation of the drug inside the cancer cell, activation of cell repair pathway, and mutations within specific drug targets causing the drug to be inefficacious.^110^ Drug resistance can be controlled by the co-delivery of drugs that have different mechanisms of action and target several pathways of cancer cell survival. Typically, ovarian cancer develops multi-drug resistance through various pathways, especially by the pump and non-pump mechanisms. The major cause of pump resistance is the overexpression of ATP-binding cassette (ABC) transporters whose role is to protect cells from toxic molecules permeating the cell by diffusion or active uptake.^110^ Non-pump resistance is facilitated by cellular antiapoptotic defense mechanism involving Bcl-2 protein which prevents cytochrome c release from mitochondria required to trigger caspase cascade for the execution of apoptosis. Use of cancer therapeutics suitable for ovarian cancer that is not substrates of ABC transporters and the employment of nanocarriers for delivery limits the challenge of drug resistance. Nanocarriers can circumvent efflux by ABC transporters as they are taken up into the cells by non-specific or specific endocytosis leading to more concentrations of loaded drugs in the cells. Co-delivery of cancer therapeutics and genes can also mitigate against multi-drug resistance. Genes such as siRNA inhibit the genes for MDR causing the tumor cells to be receptive to cancer therapeutics. Silencing of the MDR genes inhibits pump and non-pump cellular mechanisms of resistance by targeting all the intracellular molecular targets.^110^ Targeting various pathways may produce a synergistic reduction of tumor and prevent tumor proliferation. To ensure the successful co-delivery of cancer therapeutics, certain factors such as affected cancer pathways, mechanisms of action, and gene profiles need to be taken into consideration, to avoid induction of new resistances and general systemic toxicity. Co-delivery of drugs and target specificity of nanocarriers enhances therapeutic outcomes and eliminates adverse effects and multi-drug resistance. Co-delivery systems used for loading cancer therapeutics into a single drug carrier result in enhanced blood stability, elevated tumor accumulation, and same site targeting of multiple agents thereby facilitating synergistic effect and invariably producing optimal anti-cancer efficacy.^110^ The efficiency of the co-delivery of cancer therapeutics is enhanced when two or more cancer therapeutics with different pharmacological mechanisms and dissimilar adverse effects are combined. The mechanisms of action of cancer therapeutics (Table 2) include targeting the cancer cells at the DNA, RNA, or protein at the molecular level, at the organelle or nucleus on a cellular level, and endothelium and extracellular matrix at the tissue level.^28^ Nanocarriers provide an effective delivery approach for combination therapy as they can be used to load multiple drugs with different physicochemical and pharmacological properties which elicit better therapeutic effects than the loaded single drugs. Nanocarriers are also the desired strategic drug carriers due to their ability to penetrate biological barriers and enhance targeting and penetration into tumors. Co-delivery of chemotherapeutics and gene(s) in one system such as hybrid nanocarriers leads to synergistic cytotoxicity because of different mechanisms of actions of the therapeutics. The therapeutic outcome of a co-delivery system depends on the concentration ratio of the drugs as the effect could be synergistic, additive, or antagonistic.

## Conclusion

 Selective targeting capabilities of targeted hybrid nanocarriers have been proven on the bench through much research to be a feasible means of combating the scourge of cancer globally. Targeted hybrid nanocarriers for co-delivery of more than one therapeutic, co-delivery of chemotherapeutics and proteins, and delivery of genes, all in a bid to achieve additive, synergistic, or enhanced effect in cancer patients especially those with metastatic cancer or multidrug resistance, could lead to a cure and possibly prevent relapse. It is a fact that there are still studies to be done to further optimize the advantages of hybrid nanocarriers and close the translational gap between preclinical and clinical studies. Further translational research should be done so that the benefits of targeted hybrid nanocarriers can be experienced in clinical settings. Ultimately, there is a huge need to develop safe targeted hybrid nanocarrier formulations with excellent efficacy for cancer treatment while on the mission of eradicating the overwhelming morbidity and mortality rate of cancer all over the world.

## Competing Interests

 There is no conflict of interest.

## Ethical Approval

 Not applicable.
